# Microbiome–host co-oscillation patterns in remodeling of colonic homeostasis during adaptation to a high-grain diet in a sheep model

**DOI:** 10.1186/s42523-020-00041-9

**Published:** 2020-07-09

**Authors:** Limei Lin, Yue Wang, Lei Xu, Junhua Liu, Weiyun Zhu, Shengyong Mao

**Affiliations:** Jiangsu Key Laboratory of Gastrointestinal Nutrition and Animal Health, Laboratory of Gastrointestinal Microbiology, National Experimental Teaching Demonstration center of Animal Science, National Center for International Research on Animal Gut Nutrition, Centre for ruminant nutrition and feed engineering technology research, Nanjing Agricultural University, Nanjing, 210095 China

**Keywords:** Microbiome–host, Remodeling, Homeostasis, Adaptation, Colon, High-grain diet

## Abstract

**Background:**

Ruminant gastrointestinal tract homeostasis deploys interactive microbiome–host metabolic communication and signaling axes to underpin the fitness of the host. After this stable niche is destroyed by environmental triggers, remodeling of homeostasis can occur as a spontaneous physiological compensatory actor.

**Results:**

In this study, 20 sheep were randomly divided into four groups: a hay-fed control (CON) group and a high-grain (HG) diet group for 7, 14, or 28 days. Then, we examined 16S rRNA gene sequences and transcriptome sequences to outline the microbiome–host co-oscillation patterns in remodeling of colonic homeostasis in a sheep model during adaptation to a HG diet. Our data revealed that with durations of an HG diet, the higher starch levels directly affected the colonic lumen environment (lower pH and higher fermentation parameters), which in turn filtered lumen-specific functional taxonomic groups (HG-sensitive and HG-tolerant taxa). The colonic epithelium then gave rise to a new niche that triggered endoplasmic reticulum stress to activate unfolded protein response, if the duration of endoplasmic reticulum stress was overlong, this process would regulate cell apoptosis (*Caspase-3*, *Caspase-8*, and *TNFRSF21*) to achieve a functional transformation.

**Conclusions:**

Our results provide a holistic view of the colonic microbial assemblages and epithelium functional profile co-oscillation patterns in remodeling of colonic homeostasis during adaptation to an HG diet in a sheep model. These findings also provide a proof of concept that the microbe**–**host collaboration is vital for maintaining hindgut homeostasis to adapt to dietary dichotomies.

## Background

Ruminant gastrointestinal tracts are inhabited by vast and diverse symbiotic microorganisms that function in an enduring mutualistic partnership that contributes to the fitness of the host [1–3]. Through their collective interaction system, microbiome oscillations affect host physiology and genomes, which results in the creation of integrated microbial sensing and metabolic functions within the host to ensure its survival in a microbially dominated world [4]. The microbiome–host homeostasis is vital to maintaining the physiological functions of the animal’s digestive tract, which orchestrates optimized absorption, animal health, and beneficial outcomes [3]. However, this homeostasis is easily shaped throughout life, including by diet, age, and antibiotic use [5–8].

After the gastrointestinal tract niche is destroyed, remodeling of homeostasis occurs as a spontaneous physiological compensatory process [9]. That said, the microbiome and host assemble anew to adapt a changed niche at any given time according to available metabolites and physical environment conditions [10, 11]. Evidence also suggests that the host gut and its microbiota undergo co-oscillations to adjust to dietary perturbations and that these co-oscillations are vital for achieving and maintaining homeostasis [4, 12]. Thus, understanding co-oscillation patterns between the microbiome and host during remodeling of homeostasis is of importance in gastrointestinal ecology.

Over the past few years, high-grain (HG) diets have been fed to ruminants to improve the animals’ productive performance and increase the economic profits of animal production [13–15]. However, an abrupt shift from a low concentration to high concentration of grains may have adverse effects on the commensal microbiota and epithelial health of the ruminants’ gastrointestinal tracts. This increases volatile fatty acid (VFA) and lactate production and may lead to a reduction in ruminal pH, increasing the risk of less efficient fiber digestibility, reduced barrier function, and the translocation of endotoxins into the systemic circulation [16–20]. Numerous studies have demonstrated that an increase in the proportion of grain in the diet causes an increase in the amount of rumen starch passing through the small intestine and flowing into the hindgut, thereby increasing hindgut fermentation and readily affecting hindgut homeostasis [21–23]. Given this condition, a better understanding of how the hindgut microbiome and host homeostasis are remodeled during adaptation to an HG diet is important for animal health and efficient nutrient use [24]. Most researchers have assessed the transient adaptation of the forestomach and the small intestine of ruminants when exposed to an HG diet [24]. Hence, this study seeks to establish in-depth the hindgut microbiome–host co-oscillation patterns in remodeling of homeostasis due to an HG diet to underpin the whole gastrointestinal tract research.

To circumvent these challenges, we used sheep with good characteristics (high adaptability and quick growth) as the animal model to analyze 16S rRNA gene sequences of the colonic microbiome and transcriptome sequences of the colonic epithelium in four groups of sheep exposed to an HG diet for various time periods up to 28 days. Our combined time-resolved analyses may help to expand the understanding of how the microbiome and host co-vary from destroyed homeostasis to remodeling of homeostasis. The increased knowledge of the mechanisms involved in these interactions will reveal opportune interventions for the prevention of metabolic disease.

## Results

### Colonic microbial metabolites

The results of colonic fermentation stoichiometry were showed in Additional file 1: Table S1, which was published previously [25]. Briefly, the colonic pH decreased linearly (*p* = 0.007) with the increasing duration of the HG diet, while the concentrations of acetate, propionate, butyrate, lactate, and total VFA increased linearly (*p* < 0.05 for all). The valerate level was lower in the HG7 group than in the CON group, but it gradually increased to the CON group level after 14 days of the HG diet (quadratic, *p* < 0.001). Increases in the duration of the HG diet also caused cubic increases in the starch content (*p* = 0.030) and isobutyrate concentration (*p* < 0.001). These metabolites were higher in the HG7 group, lower in the HG14 group, and higher in the HG28 group (cubic, *p* < 0.05), whereas duration of the HG diet had no effect on the concentration of isovalerate (*p* > 0.05).

An increasing duration of HG feeding had no effect on the proportion of acetate. The percentages of propionate (*p* = 0.007) and valerate (*p* < 0.001) were lower in the HG7 group than in the CON group, but they gradually increased with adaptation to the HG diet. A linear decrease was detected in the molar proportions of isobutyrate (*p* < 0.001) and isovalerate (*p* < 0.001) with duration of the HG diet, while the proportion of butyrate (*p* < 0.001) increased linearly.

### The diversity and richness of colonic bacterial communities

**Fig. 1:**
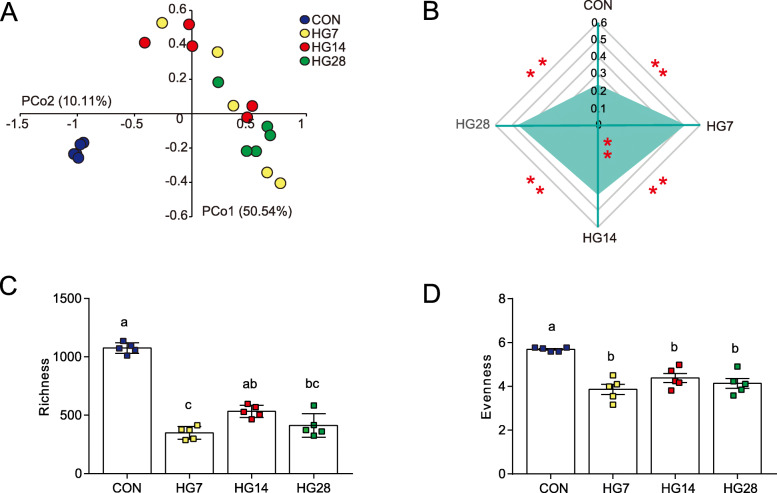
(A) PCoA analysis of bacterial communities in the colonic digesta samples among 20 sheep assigned to CON (*n* = 5), HG7 (*n* = 5), HG14 (*n* = 5), and HG28 (*n* = 5) groups, which received an HG diet for 0, 7, 14, and 28 days, respectively. (B) Comparisons of the distances based on the unweighted UniFrac dissimilarity matrix among the four groups at the OTU level. *p < 0.05, **p < 0.01. (C) The colonic bacterial richness (number of observed species) and (D) evenness (Shannon diversity index values) at the 3% dissimilarity level

Across all 20 samples, a total of 683,600 high-quality reads and an average of 34,180 ± 4085 reads per sample were obtained with 16S rRNA gene sequencing. As shown in Additional file 2: Fig. S1, the rarefaction curves approached a plateau at 26,706 reads. The results of principal co-ordinate analysis (PCoA) based on an unweighted UniFrac distance revealed that the colonic microbial structures were distinctly separate from each other (Fig. 1A). An unweighted distance-based analysis of molecular variance (AMOVA) based on the unweighted distance metric (Additional file 3: Table S2) verified statistically significant dissimilarities among the four groups with respect to bacterial diversity (*F* = 6.384; *p* < 0.001). Based on unweighted UniFrac dissimilarity matrix among the four groups at the OTU level, we used a dissimilarity metric to measure the resilience of bacterial communities (Fig. 1B). Of note, the community diversity of the HG14 group had the closest metric among the HG groups (*p* < 0.01), but there was still a significant difference between the HG14 and HG28 groups. We further compared the α-diversity across the 20 samples. As shown in Fig. 1C and D, bacterial richness (operational taxonomic unit [OTU] numbers; *p* = 0.002) and evenness (Shannon index; *p* = 0.006) followed a similar pattern of change, surprisingly presenting a reverse trend compared with intragroup metrics among the four groups. Specifically, all sheep fed an HG diet had lower index values than the control hay-fed sheep, and reached a peak at 14 days of HG feeding only for bacterial richness. All indices are shown in Additional file 4: Table S3.

### Taxonomic configurations of colonic bacteria during adaptation to the HG diet

**Fig. 2:**
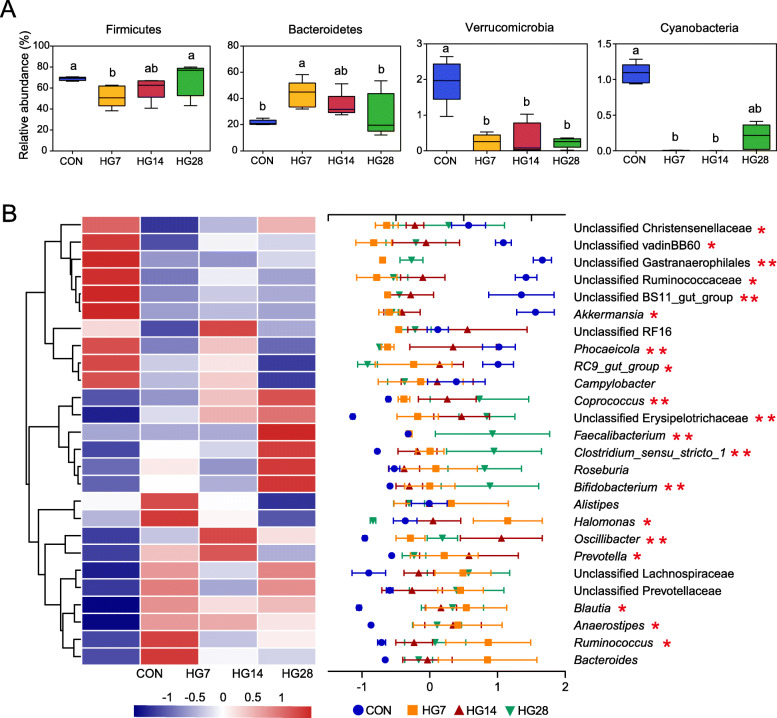
Dominant phyla or taxa of bacteria that had a relative abundance of more than 1% for at least one group were compared among the four groups. (A) Phylum-level differences in relative abundance among four groups. (B) Distribution of the predominant taxa in colonic digesta. The color key of the heat map depicts the z-score of the relative abundance of the dominant taxa. *p < 0.05, **p < 0.01

As a percent of reads assigned, discriminatory features were evident in the bacterial relative abundance at both the phylum and genus levels. The threshold was identical, as each taxonomic configuration was more than 1% of the mean relative abundance for at least one group. We also distinguished between HG-sensitive and HG-tolerant indicators, which decreased and increased, respectively, in response to an HG diet. At the phylum level (Additional file 5: Table S4), Firmicutes, Bacteroidetes, and Proteobacteria were the most abundant, representing more than 93% of the bacterial community (93.46% in the CON group, 98.55% in the HG7 group, 98.06% in the HG14 group, and 96.99% in the HG28 group). With increasing durations of HG feeding, significant shifts were noted in four phyla: Firmicutes, Bacteroidetes, Verrucomicrobia, and Cyanobacteria (*p* < 0.05; Fig. 2A). Of these, Firmicutes was sensitive to the initial dietary perturbation from hay to the HG diet, and it then increased with the duration of the HG diet. However, Bacteroidetes was an indicator of HG tolerance. Additional bacterial HG-sensitive indicators were found in the Verrucomicrobia and Cyanobacteria.

At the genus level, a heat map based on the z-score of the relative abundance (≥ 1% in at least one group) of 26 predominant taxa was prepared to determine the distribution of taxonomic composition in colonic digesta among the four groups (Fig. 2B). Of the 19 predominant taxa that significantly shifted during this study (*p* < 0.05; Additional file 6: Table S5), the relative abundance of *Blautia*, *Coprococcus*, *Ruminococcus*, *Oscillibacter*, *Clostridium_sensu_stricto_1*, unclassified Erysipelotrichaceae, *Anaerostipes*, *Prevotella*, and *Bifidobacterium* were tolerant to HG feeding, and their proportions remained stable in all three HG groups. Similarly, the proportions of unclassified Ruminococcaceae, unclassified Christensenellaceae, unclassified vadinBB60, *RC9_gut_group*, unclassified BS11_gut_group, *Akkermansia*, and unclassified Gastranaerophilales were lower in the HG groups compared to the CON group, and no differences in the proportions of these taxa were found between the HG groups. Over the entire experimental period, the relative abundance of some taxa changed dynamically and continuously. For example, the abundance of *Faecalibacterium* was stable in the CON, HG7, and HG14 groups, but showed a significant increase in the HG28 group. *Phocaeicola* decreased from the CON group to the HG7 group but increased in the HG14 group, followed by a decrease in the HG28 group. No significant difference was detected in the relative abundance of *Halomonas* in the CON, HG14, and HG28 groups, while the percentage of *Halomonas* was higher in the HG7 group than in the HG28 group.

### Gene co-expression networks from dietary perturbation to duration of HG diet

**Fig. 3:**
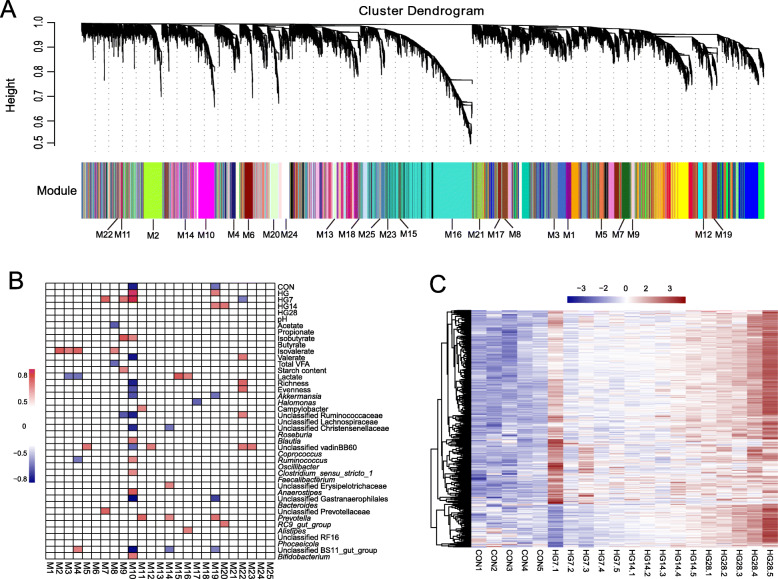
Global co-expression networks and gene modules. **a** Dendrogram from gene co-expression network analysis of samples among the CON, HG7, HG14, and HG28 groups. Modules of co-expressed genes were assigned a color and number (M1 to M25). **b** Relationship between dietary factors, durations, fermentation parameters, bacterial traits, and the 25 gene modules. Only Spearman’s significance levels (*p* < 0.05, *r* ≥ 0.5) are shown. **c** Heat map of genes in M10 showing the dynamic expression pattern after hierarchical clustering among the four groups. The color key of the heat map depicts the z-score of the expression of genes in M10

We generated 152.90 Gb of clean data for the host gene transcription, with an average of 7.65 Gb (± 0.20 SEM) of clean data per subject. In total, the expression profiles of 13,206 genes with fragments per kilobase of transcript per million fragments mapped (FPKM) > 0.2 were determined in all colonic tissue samples. To obtain significant bioinformatics in the multidimensional transcriptome profiles, we performed weighted gene co-expression network analysis (WGCNA) to identify modules of co-expressed genes. We divided the common host genes into 25 modules (named M1**–**M25) (Fig. 3a).

### Functional profiles of microbiome-associated host transcriptome modules

**Fig. 4:**
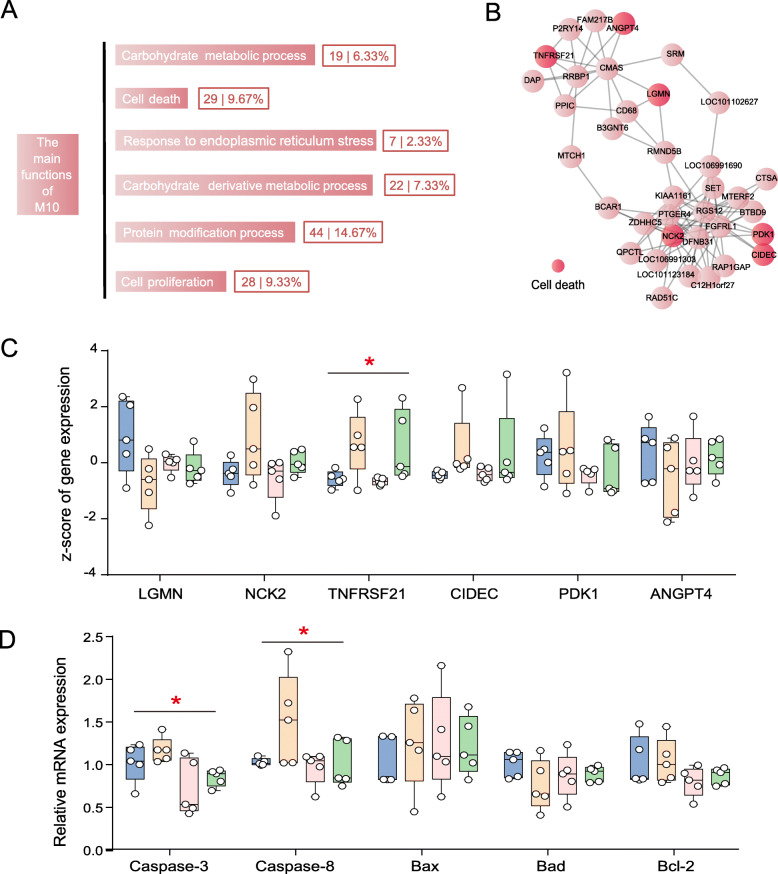
**a** The significant GO terms (*p* < 0.05) in biological processes of the M10. **b** The network of hub genes in the M10. The six genes in red were enriched in a cell apoptotic process. **c** The z-sore of six gene expressions, including *LGMN*, *NCK2*, *INFRSF21*, *CIDEC*, *PDK1*, and *ANGPT4*, are shown among the four groups based on transcriptome analysis. *p < 0.05. **d** The relative mRNA abundance of genes related to cell apoptosis by qRT-PCR in the colon of hay-fed (CON) and HG-diet-fed sheep (HG7–28). Values are means (*n* = 5), with their standard errors represented by vertical bars

To mechanistically probe the interaction between the microbiome and host, we used observed indexes (dietary factors, durations, fermentation parameters, and bacterial traits) to associate colon epithelium expressed modules. Among the 25 modules, the expression of host genes in the M10 module (442 genes; 3.35% of total reads) had the strongest correlation with microbial traits (Fig. 3b). Notably, these genes in the M10 module displayed positive associations with the high-concentration diet and negative associations with low-concentration diet. The concentration of several VFAs was also closely related to M10. Not surprisingly, the M10 module was also positively correlated with HG-tolerant taxa indicators but negatively associated with HG-sensitive taxa and diversity indices. These results imply the shift of diet wound trigger epithelial genes expression. Macroscopically, we also identified that the M10 module was a hallmark of the oscillation pattern, which was coordinated with a shift in the bacterial community (Fig. 3c). To identify M10-related functions, we conduct Gene Ontology (GO) enrichment analysis of the biological processes. Only GO terms with *p* < 0.05 were regarded as significantly regulated (Fig. 4a; Additional file 7: Table S6). Among 50 significantly altered GO terms, we summarized main functions, including carbohydrate metabolic process (6.33%), cell death (9.67%), response to endoplasmic reticulum stress (ERS) (2.33%), carbohydrate derivative metabolic process (7.33%), protein modification process (mainly unfolded protein response [UPR]; 14.67%), and cell proliferation (9.33%).

### Expression trajectories of M10 hub genes

Using the MCODE application in Cytoscape (version 3.5.1), we found that M10 was mainly conducted by 36 hub genes (Fig. 4b; Additional file 8: Table S7)—including *LGMN*, *NCK2*, *TNFRSF21*, *CIDEC*, *PDK1*, and *ANGPT4*—which have been mainly implicated in the cell apoptotic process in the colonic epithelium. Among them, the expression trajectories of *TNFRSF21* showed significant oscillations due to dietary perturbations and duration of HG diet (*p* = 0.042; Fig. 4c). These results imply that the microbiome may manipulate the host transcriptome by regulating cell apoptosis. To further investigate, we also quantified the relative mRNA abundance of genes related to cell apoptosis function (*Caspase-3*, *Caspase-8*, *Bcl-2*, *Bax*, and *Bad*) by qRT-PCR in the colons of hay-fed (CON) and HG-fed sheep (HG7, HG14, and HG28). Intriguingly, drastic expression shifts were found in *caspase-3* (*p* = 0.025) and *caspase-8* (*p* = 0.046); of note, the oscillatory trend of *caspase-3* and *caspase-8* longitudinally coincided with M10 hub genes enriched in cell apoptosis (Fig. 4d). Additionally, the expressions of these three shifts in pro-apoptosis genes was the highest in the HG7 group, and then had attenuated trend with the duration of HG feeding.

## Discussion

The objective of this study was to understand the co-diversified patterns between taxonomic configurations and colonic transcriptome files in remodeling of colonic homeostasis during adaptation to an HG diet in a sheep model. We designed our experiment to follow the main events from a high-forage to a high-starch diet and then longitudinal HG feeding for 4 weeks. Not surprisingly, after abruptly switching to an HG diet, the colonic niche underwent comprehensive modification that included increased dietary starch content, enhanced total VFA production, promoted lactate, and decreased pH. These results were also mirrored by our previous study in rumen, which described a hallmark of the significant increased concentrations of ruminal butyrate, valerate, lactate and total VFA in the HG groups compared to their levels in the CON group [26]. Additionally, the high-starch diet drove clearly distinguishable changes in colon microbial ecosystems, and the duration of the HG-diet regulated the divergence and convergence of diet-microbe fingerprints in this study. These observations were followed the previously described natural oscillations in rumen [27, 28]. That said, ruminal microbial community structure and composition would be changed and adapted from a high-forage diet to this to a high-grain diet HG. To explore whether the changes in the rumen and colon lumen environment are convergent, we further studied the changes in the colonic microbial ecology with high comprehensive. Considering that the colonic microbial structures were distinctly separated from each other using an unweighted UniFrac distance, we also analyzed inter-individual variability matrix among the four groups at the OTU level. One intriguing phenomenon of the microbial dissimilarity metric was that the community diversity of the CON group had the minimal inter-individual variability while the HG7 group had maximum inter-individual variability, which implies that the HG diet disturbed the microbial niche [1, 27]. After feeding with an HG diet, the HG14 group showed the greatest similarity, but there still had significantly similarities between HG14 and HG28 groups. That said, the microbial structure always assembled anew to adapt to the distinct colonic niches throughout the duration of HG feeding. We further compared α-diversity among the four groups and found that bacterial richness and evenness followed a similar pattern of change, with the sudden switch to an HG diet resulting in lower index values than the hay-fed sheep. The more striking observation from our study was the reverse trend compared with intragroup metrics among the four groups, which is in agreement with previous ruminal observations [28, 29]. This implies that dissimilarity based on an unweighted UniFrac distance and bacterial richness and evenness of α-diversity may be indicators of microbial homeostasis disturbed by dietary perturbation.

After observing that an HG diet drove a colonic niche shift, we could see extreme changes in distant phylogenetic lineages moving up the taxonomic levels of phyla and genera, which distinguished between HG-sensitive and HG-tolerant indicators. Phylogenetic analysis of detectable microbial genera revealed a change in relative abundance in four phyla, namely Firmicutes, Bacteroidetes, Verrucomicrobia, and Cyanobacteria. Notably, opposite trends were observed between Firmicutes and Bacteroidetes. Their relative abundances shifted significantly when faced with dietary perturbation. For Firmicutes, which was significantly decreased when abruptly feeding HG diet, while constantly increased with the increased duration of the HG diet. Firmicutes has been reported to perform a starch-degradation function [16], which hints the increased Firmicutes adapted to continuous HG diet. Contrarily, the levels of Bacteroidetes increased primordially, then decreased with the duration of the HG diet being sensitive to high levels of starch. This observation had a corresponding increase in high starch content. These results illustrate that the microbiome was able to remodel its homeostasis ability to adapt to new habitats within a certain period of time, selecting and shaping specific functional groups according to physical environmental landscapes during continuous HG feeding.

We also observed that the changes in taxa showed remodeling oscillations at the genus level. Noteworthy features were three oscillatory patterns throughout the various duration times of the HG diet. Among them, nine taxa were HG-tolerant indicators, as demonstrated by their increased tendency after HG feeding, and their proportions remained stable for the duration of the HG diet. *Prevotella* participates in the digestion and utilization of starch, xylan, and pectin in ruminants, and so its higher abundance in the HG groups might be due to the higher level of starch, which favors the growth of this diverse functional contributor in the colonic bacterial community [30, 31]. Similarly, *Oscillibacter* has a positive relationship with the starch content in cattle feces, so the higher percentage of this genus observed in the HG groups may reflect the high levels of fermentable substrate in the sheep’s colon [32]. Additionally, *Bifidobacterium* is considered a starch-hydrolyzing bacteria [33]. The increases in *Coprococcus*, which results in increased production of butyrate, may have given rise to a higher-starch niche to provide energy to the host [34, 35]. The higher relative abundance of *Blautia* could increase fermentation to produce more lactate and acetate, as described previously [36]. These increases may be accompanied by an increase in the abundance of specific functional groups associated with starch breakdown and fermentation, which lead to the colonic pH drop caused by the production of multiple metabolites (organic and short-chain fatty acids) [19]. This phenomenon hints that the colonic bacterial community has different properties but performs consistent functions in response to an HG diet.

An opposite trend was seen with the unclassified Ruminococcaceae, unclassified Christensenellaceae, unclassified vadinBB60, *RC9_gut_group*, unclassified BS11_gut_group, *Akkermansia*, and unclassified Gastranaerophilales. These taxa characterized the colonic microbiome of the CON group, and the duration of the HG diet had no effect on the proportion of these taxa. For example, the decreases in hydrogen-producing fiber-degrading unclassified Christensenellaceae [37], hemicellulose-degradation unclassified BS11_gut_group [38], and forage-enrichment *Akkermansia* [32] after HG feeding implies that the microbiome has an abridged response to dietary perturbations when switching from high-forage to high-starch diets. Another trait of the oscillating remodeling pattern is that the relative abundance of some taxa changed dynamically and continuously with the duration of the HG diet. We found the abundance of *Faecalibacterium* remained stable in the CON group, the HG7 group, and the HG14 group, but a significant increase was observed in the HG28 group. Interestingly, *Faecalibacterium* may use substrates to ferment more butyrate [23]. This genus was accompanied by a corresponding increase in the percentage of *Coprococcus* to produce more butyrate, which may explain the highest butyrate concentration in the HG28 group. Taken together, the changes in the functional taxonomic groups were affected by the high-starch niche throughout the HG feeding period. This suggests that the functional taxonomic groups, regardless of whether they are HG-sensitive or HG-tolerant, and microbial metabolites together played a vital role in stabilizing the microbial ecosystem throughout the HG feeding period.

Evidence now supports cross-talk between the microbiota genomes and the host genome, and the most important regulatory factor in this process is diet [6]. In this study, we used the transcriptome profile to perform WGCNA to identify modules of co-expressed genes associated with the enteric environment (dietary factors, fermentation parameters, and microbial-specific assemblages). Not surprisingly, we found that the M10 module had co-diversified with various traits. Notably, the M10 module, a hallmark of the oscillation pattern, was positively correlated with HG-tolerant taxa indicators and negatively associated with HG-sensitive taxa and diversity indices. These relationships and oscillation patterns suggest that dietary dichotomy and the temporal variation in feeding of an HG diet caused a convergence between microbial traits and available host transcriptome files, which together restored homeostasis to new habitats (HG diet) [39].

To further elucidate the functional mechanisms behind colonic microbiome–host co-oscillation patterns during adaptation to an HG diet, we summarized the main functions of M10, including the carbohydrate metabolic process, cell death, response to ERS, carbohydrate derivative metabolic process, protein modification process (mainly UPR), and cell proliferation. According to previous observations, we found that the endoplasmic reticulum is an organelle with multiple functions that participates in transmembrane protein synthesis and secretion [40]. When facing metabolic disturbance stemming from over nutrition, it aggravates intracellular stress (ERS) [41]. ERS triggers the UPR to restore homeostasis (protein modification process); if the adaptive response fails, cell apoptosis ensues [40, 42]. Interestingly, these series of previous events were highly mirrored by our findings; the sheep who underwent a dietary change from a high-forage diet to HG diet demonstrated intracellular niche shifts that then gave rise to cell apoptosis. Therefore, we posit a tantalizing hypothesis that during adaptation to an HG diet, the diet microbiome drove host transcriptome files shifts, potentially through regulating cell apoptosis.

We further analyzed the highest degree of connectivity genes belonging to M10 and found that 36 hub genes of M10 were mainly implicated in cell apoptotic process function in the colonic epithelium. Among them, the expression trajectories of *TNFRSF21* from dietary perturbations to duration of HG diet had significant oscillation. Previous evidence shows that *TNFRSF21*, belonging to ERS and proinflammation, induces cell apoptosis when facing environmental stress [43, 44]. To our understanding, these findings highlight cell apoptosis as the main driver for its host transcriptome oscillations. To further investigate this, we also quantified the relative mRNA abundance of other genes related to cell apoptosis among the four groups. Intriguingly, drastic expression shifts were found in *Caspase-3* and *Caspase-8*, in which the oscillatory trends longitudinally coincided with M10 hub genes enriched in cell apoptosis. Under ERS-inducing stimulation, the caspase family of cysteine proteases involved in apoptosis and inflammatory cytokine, such as *caspase-3* and *caspase-8*, was activated [40, 41]. These results show that cell apoptosis was the highest after the sudden shift from a high-forage to HG diet, and the pro-apoptosis process was attenuated throughout the duration of feeding with the HG diet. That said, microbiome-driven host cell apoptosis was the main functional driver for regulating colonic epithelium transcriptome remodeling oscillations to adapt to the HG environment.

## Conclusions

**Fig. 5:**
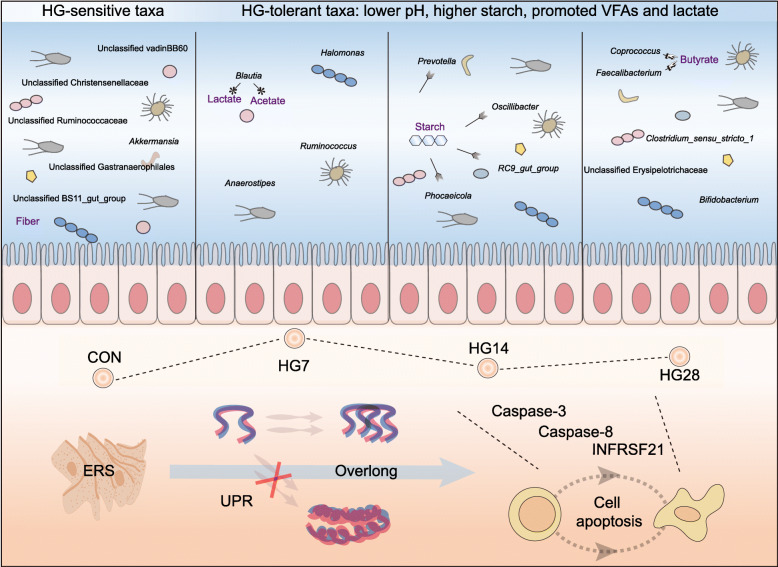
A holistic view of microbiome–host co-oscillation patterns in remodeling of colonic homeostasis during adaptation to an HG diet in a sheep model

Generally, the increased level of starch directly affected the colonic lumen environment as the duration of the HG diet increased, which in turn filtered the lumen-specific functional taxonomic groups (Fig. 5). The colonic epithelium then gave rise to a new niche that triggered cell apoptosis to achieve a functional transformation. These finding revealed that microbe–host collaboration is vital for remodeling of hindgut homeostasis to allow adaptation to dietary perturbations.

## Methods

### Animals

This study was approved by the Nanjing Agricultural University in compliance with the Regulations for the Administration of Affairs Concerning Experimental Animals. The detailed design of the animal experiments was the same as in our previous study [25]. Briefly, we selected 20 male Hu sheep (180 days old with a body weight of 25.60 ± 0.41 kg) and randomly divided them into four groups (CON, HG7, HG14, and HG28), with five animals in each group. Before the study began, all sheep were fed a hay-based diet (Additional file 9: Table S8) for 4 weeks to build a similar colon environment. After the transition period, the sheep from the CON group continued to be fed hay for 28 days, while the three HG groups received a total mixed ration having a 60:40 concentrate-roughage ratio (Additional file 9: Table S8; concentrate containing corn grain, wheat grain, soybean meal, mineral and vitamin premixes, and roughage including alfalfa hay and oat hay) for 7, 14 and 28 days. All animals were fed 3.50% of their body weight per day and placed in individual pens (1.2 × 1.4 m) with free access to water. The animals were fed twice a day at 0830 and 1630 h, and there was approximately 10% feed refusal. The body weight of the sheep was measured on the first day of every week before feeding.

### Sample collection and measurements

Animals from each treatment group were slaughtered and sampled on the day of their feeding treatment completion. Digesta samples in the colon were collected immediately after dissection, and 7 ml of homogenized colonic content samples were collected and preserved in liquid nitrogen for DNA extraction. Colon tissue was washed with ice-cold phosphate-buffer saline and cut into 0.4 × 0.4-cm strips using a scalpel, then preserved in liquid nitrogen for RNA extraction.

### Extraction of bacterial DNA from colonic digesta

We extracted microbial DNA from 0.25 g of each colonic digesta sample using a DNA extraction kit (QIAamp Fast DNA Stool Mini Kit, Germany). The concentration and quality of the DNA was determined with a Nanodrop 1000 spectrophotometer (Thermo Fisher Scientific, USA), and the DNA was preserved at − 80 °C for subsequent analyses.

### PCR amplification and 16S rRNA gene sequencing

The bacterial 16S rRNA gene was amplified from isolated colon digesta DNA using the typical 338F-806R primers (V3–V4 region). The qualified DNA was broken into libraries and sequenced using the Illumina PE250 MiSeq platform. The paired reads generated from the platform were then checked using the previously described criteria for quality control [45]. High quality sequences with a threshold sequence similarity level great than 97% were clustered into OTUs using UPARSE software (version 7.1; http://drive5.com/uparse/) [46]. We selected the sequences with the maximum abundance in each OTU as representative sequences using QIIME (version 1.9.0) software [47]. The representative sequences of each OTU were compared to the SILVA database (version 119) [48]. Rarefaction curves, alpha diversity, beta diversity and Good’s coverage were calculated in QIIME using the default parameters. AMOVA of the unweighted UniFrac distance was performed using MOTHUR software [49].

### Epithelial RNA extraction

We ground the colon tissues into powder and extracted total RNA using TRIzol (Takara Bio, Otsu, Japan) according to a previously described method [50]. The concentration and quality of total RNA were determined with a Nanodrop 1000 spectrophotometer. The absorbed optical density ratio (OD260/280) for RNA was selected to maintain a high RNA purity (1.85–2.05), and the RNA integrity was verified by 1.4% agarose-formaldehyde gel electrophoresis. The concentration of each RNA sample was normalized to 500 ng/μL for each sample based on optical density, and each sample was preserved at − 80 °C for subsequent analyses. Then, 1 μg of RNA was used for sequencing, sequencing libraries were generated using a NEBNext Ultra RNA Library Preparation Kit (E7530L, NEB, USA) following the manufacturer’s recommendations for Illumina Hiseq 2000 platform, and index codes were added to attribute sequences to each sample. Another 1 μg of RNA was reverse-transcribed using a PrimeScript® RT reagent Kit with a gDNA Eraser (Takara Bio, Shiga, Japan). The primer sets used in our research were listed in Additional file 10: Table S9.

### Transcriptome sequencing and analysis

After obtaining the paired-end reads, we used the internal script to remove low-quality reads. HISAT2 (www.ccb.jhu.edu/people/infphilo) was used to align the remaining reads to the host [51]. The software StringTie (version 1.3.4d) was used to map reads in order to calculate the expression of transcripts using FPKM methods [52]. Only expressed genes (FPKM > 0.2 in all samples) were used in subsequent analyses. The GO enrichment of genes was analyzed using DAVID (version 6.8) [53].

### Statistical analyses

Data for the microbial traits obtained from 16S rRNA gene sequencing and differentially expressed genes (DEGs) based on transcriptome analysis were analyzed using the Kruskal–Wallis test to identify significant shifts based on threshold values (*p* < 0.05). Additionally, apoptosis-related genes were quantified by qRT-PCR, which was used to perform differential calculation with one-way ANOVA. The distribution of the predominant genera, the relationship between colonic expressed modules and microbial traits, and expression trajectories of M10 hub genes in colonic digesta among the four groups were visualized by using the “pheatmap” package in the R software (version 3.5.0). The WGCNA tool in the R package was used to construct a gene co-expression network and associate modules, dietary factors, durations, fermentation parameters, and bacterial traits [54]. The gene network was exported to Cytoscape for visualization [55]. Modular structure and groups of highly interconnected nodes were analyzed using the MCODE application in Cytoscape with standard parameters (node score cutoff: 0.2; K-Core: 2; maximum depth from seed: 100) [56].

## Supplementary information

**Additional file 1 Table S1.** Serial changes in the colonic fermentation stoichiometry (Mean values with their standard errors; *n* = 5). CON, 0 day fed an HG diet; HG7, 7 days fed an HG diet; HG14, 14 days fed an HG diet; HG28, 28 days fed an HG diet.

**Additional file 2 Figure S1.** The rarefaction curves of the colonic digesta of hay-fed (CON) and concentrate-fed sheep (HG7–28).

**Additional file 3 Table S2.** Analysis of molecular variance (AMOVA) of bacterial communities in colonic digesta of hay-fed (CON) and concentrate-fed sheep (HG7–28).

**Additional file 4 Table S3.** Serial changes in the richness and diversity of colonic bacterial community.

**Additional file 5 Table S4.** Serial changes in the abundance of predominant phyla (% of total sequences) in colonic digesta. (Mean values with their standard errors; *n* = 5).

**Additional file 6 Table S5.** Serial changes in the abundance of predominant genera (% of total sequences) in colonic digesta. (Mean values with their standard errors; *n* = 5).

**Additional file 7 Table S6.** GO enrichment analysis of the M10 genes in biological processes. Only terms with *p* < 0.05 were listed.

**Additional file 8 Table S7.** The expression of hub genes was enriched in the M10 module.

**Additional file 9 Table S8.** Ingredient and chemical composition of the diet (dry matter basis).

**Additional file 10 Table S9.** The primer sequences of genes related to cell apoptosis in the colonic epithelium for qRT-PCR.

## Data Availability

The 16S rRNA gene sequencing data for all the samples analysed in this study were submitted to the National Centre for Biotechnology Information Sequence Read Archive (SRA) under accession SRP106824, BioProject PRJNA386096. The transcriptome analysis data for all samples described above were deposited into the NCBI Sequence Read Archive (SRA) database (BioProject PRJNA533714 and SRA Study SRP193635).

## Abbreviations

HG: High-grain
VFA: Volatile fatty acid
PCoA: Principal co-ordinate analysis
AMOVA: An unweighted distance-based analysis of molecular variance
OTU: Operational taxonomic unit
FPKM: Fragments per kilobase of transcript per million fragments mapped
GO: Gene Ontology
DAVID: The Database for Annotation, Visualization and Integrated Discovery
WGCNA: Weighted gene co-expression network analysis
DEGs: Differentially expressed genes
ERS: Endoplasmic reticulum stress
UPR: Unfolded protein response
