# Supplementary material for: Microbiome–host co-oscillation patterns in remodeling of colonic homeostasis during adaptation to a high-grain diet in a sheep model
Source: Anim Microbiome. 2020 Jul 9;2:22. doi: 10.1186/s42523-020-00041-9 (PMC7807687; doi:10.1186/s42523-020-00041-9)
Supplement: Supplementary file 1 — Additional file 1 Table S1. Serial changes in the colonic fermentation stoichiometry (Mean values with their standard errors; n = 5). CON, 0 day fed an HG diet; HG7, 7 days fed an HG diet; HG14, 14 days fed an HG diet; HG28, 28 days fed an HG diet. [file 42523_2020_41_MOESM1_ESM.docx]

**Table S1. Serial changes in the colonic fermentation stoichiometry (Mean values with their standard errors; n=5).** CON, 0 day fed an HG diet; HG7, 7 days fed an HG diet; HG14, 14 days fed an HG diet; HG28, 28 days fed an HG diet**.**

| Items | CON | HG7 | HG14 | HG28 | SEM | *P*-value | | | |
| --- | --- | --- | --- | --- | --- | --- | --- | --- | --- |
|  |  |  |  |  |  | CON vs HG | Linear | Quadratic | Cubic |
| pH | 6.70 | 6.55 | 6.52 | 6.11 | 0.080 | <0.001 | 0.007 | 0.339 | 0.462 |
| Acetate umol/g | 21.48 | 35.97 | 38.37 | 51.59 | 2.645 | <0.001 | <0.001 | 0.781 | 0.041 |
| Propionate umol/g | 3.70 | 3.64 | 4.50 | 6.60 | 0.354 | <0.001 | <0.001 | 0.049 | 0.902 |
| Isobutyrate umol/g | 0.42 | 0.65 | 0.33 | 0.61 | 0.033 | <0.001 | 0.154 | 0.597 | <0.001 |
| Butyrate umol/g | 1.03 | 3.17 | 5.02 | 6.27 | 0.507 | <0.001 | <0.001 | 0.395 | 0.903 |
| Isovalerate umol/g | 0.24 | 0.22 | 0.16 | 0.18 | 0.018 | <0.001 | 0.111 | 0.600 | 0.496 |
| Valerate umol/g | 0.25 | 0.03 | 0.17 | 0.28 | 0.024 | <0.001 | 0.022 | <0.001 | 0.001 |
| Total VFA umol/g | 27.12 | 43.68 | 48.56 | 65.49 | 3.343 | <0.001 | <0.001 | 0.944 | 0.069 |
| Starch content % of DM | 0.48 | 2.47 | 0.53 | 1.29 | 0.003 | 0.006 | 0.851 | 0.325 | 0.030 |
| Lactate umol/g | 2.30 | 2.39 | 3.02 | 3.45 | 0.190 | <0.001 | 0.015 | 0.633 | 0.657 |
| Percent of acetate % | 79.18 | 82.15 | 78.92 | 78.82 | 0.634 | <0.001 | 0.426 | 0.224 | 0.116 |
| Percent of propionate % | 13.65 | 8.52 | 9.31 | 10.05 | 0.626 | <0.001 | 0.028 | 0.007 | 0.203 |
| Percent of isobutyrate % | 1.54 | 1.51 | 0.69 | 0.93 | 0.092 | <0.001 | <0.001 | 0.188 | 0.001 |
| Percent of butyrate % | 3.80 | 7.22 | 10.38 | 9.49 | 0.648 | <0.001 | <0.001 | 0.006 | 0.248 |
| Percent of isovalerate % | 0.90 | 0.54 | 0.34 | 0.27 | 0.075 | 0.288 | <0.001 | 0.190 | 0.958 |
| Percent of valerate % | 0.93 | 0.06 | 0.36 | 0.43 | 0.075 | 0.547 | 0.001 | <0.001 | <0.001 |
