# Supplementary figures and images for: Microbiome–host co-oscillation patterns in remodeling of colonic homeostasis during adaptation to a high-grain diet in a sheep model
Source: Anim Microbiome. 2020 Jul 9;2:22. doi: 10.1186/s42523-020-00041-9 (PMC7807687; doi:10.1186/s42523-020-00041-9)

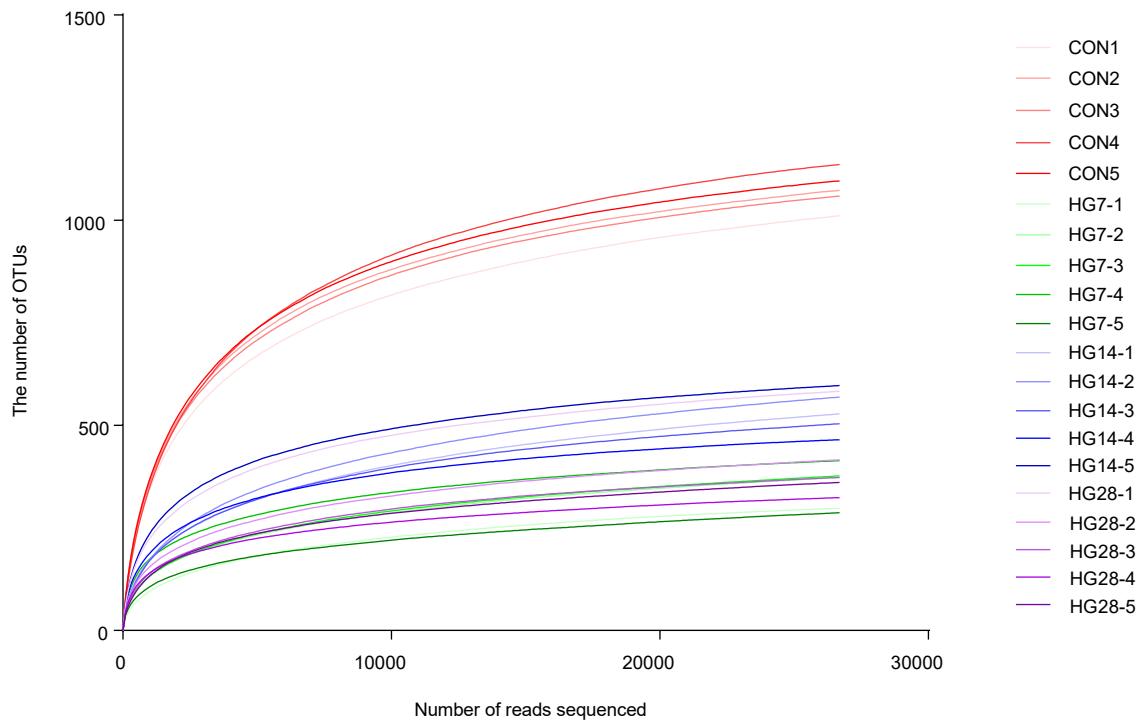

Supplement: Supplementary file 2 — Additional file 2 Figure S1. The rarefaction curves of the colonic digesta of hay-fed (CON) and concentrate-fed sheep (HG7–28). [file 42523_2020_41_MOESM2_ESM.pdf]
