# Supplementary material for: Microbiome–host co-oscillation patterns in remodeling of colonic homeostasis during adaptation to a high-grain diet in a sheep model
Source: Anim Microbiome. 2020 Jul 9;2:22. doi: 10.1186/s42523-020-00041-9 (PMC7807687; doi:10.1186/s42523-020-00041-9)
Supplement: Supplementary file 3 — Additional file 3 Table S2. Analysis of molecular variance (AMOVA) of bacterial communities in colonic digesta of hay-fed (CON) and concentrate-fed sheep (HG7–28). [file 42523_2020_41_MOESM3_ESM.docx]

**Table S2. Analysis of molecular variance (AMOVA) of bacterial communities in colonic digesta of hay-fed (CON) and concentrate-fed sheep (HG7–28).**

|  | CON | HG7 | HG14 | HG28 |
| --- | --- | --- | --- | --- |
| CON |  |  |  |  |
| HG7 | 0.006 |  |  |  |
| HG14 | 0.012 | 0.010 |  |  |
| HG28 | 0.009 | 0.048 | 0.006 |  |
