# Supplementary material for: Microbiome–host co-oscillation patterns in remodeling of colonic homeostasis during adaptation to a high-grain diet in a sheep model
Source: Anim Microbiome. 2020 Jul 9;2:22. doi: 10.1186/s42523-020-00041-9 (PMC7807687; doi:10.1186/s42523-020-00041-9)
Supplement: Supplementary file 4 — Additional file 4 Table S3. Serial changes in the richness and diversity of colonic bacterial community. [file 42523_2020_41_MOESM4_ESM.docx]

**Table S3. Serial changes in the richness and diversity of colonic bacterial community.**

| Items | CON | HG7 | HG14 | HG28 | SEM | *P* |
| --- | --- | --- | --- | --- | --- | --- |
| OTU | 1075^a^ | 350^c^ | 533^ab^ | 411^bc^ | 67 | 0.002 |
| ACE | 1190^a^ | 435^c^ | 623^ab^ | 483^bc^ | 71 | 0.002 |
| Chao 1 | 1199^a^ | 424^c^ | 630^ab^ | 495^bc^ | 72 | 0.002 |
| Shannon | 5.69^a^ | 3.86^b^ | 4.38^b^ | 4.13^b^ | 0.184 | 0.006 |
| Simpson | <0.01^b^ | 0.51^a^ | 0.43^a^ | 0.48^a^ | 0.006 | 0.013 |

(Mean values with their standard errors; n=5)
