# Supplementary material for: Microbiome–host co-oscillation patterns in remodeling of colonic homeostasis during adaptation to a high-grain diet in a sheep model
Source: Anim Microbiome. 2020 Jul 9;2:22. doi: 10.1186/s42523-020-00041-9 (PMC7807687; doi:10.1186/s42523-020-00041-9)
Supplement: Supplementary file 5 — Additional file 5 Table S4. Serial changes in the abundance of predominant phyla (% of total sequences) in colonic digesta. (Mean values with their standard errors; n = 5). [file 42523_2020_41_MOESM5_ESM.docx]

**Table S4. Serial changes in the abundance of predominant phyla (% of total sequences) in colonic digesta. (Mean values with their standard errors; n = 5)**

| Phylum | CON | HG7 | HG14 | HG28 | SEM | *P* |
| --- | --- | --- | --- | --- | --- | --- |
| Firmicutes | 68.91^a^ | 52.24^b^ | 59.83^ab^ | 68.07^a^ | 2.701 | 0.040 |
| Bacteroidetes | 21.65^b^ | 43.15^a^ | 34.68^ab^ | 27.43^b^ | 2.899 | 0.034 |
| Proteobacteria | 2.90 | 3.16 | 3.55 | 1.49 | 0.380 | 0.224 |
| Actinobacteria | 1.20 | 0.92 | 0.52 | 1.95 | 0.293 | 0.287 |
| Verrucomicrobia | 1.95^a^ | 0.23^b^ | 0.34^b^ | 0.23^b^ | 0.186 | 0.016 |
| Cyanobacteria | 1.08^a^ | <0.01^b^ | <0.01^b^ | 0.20^ab^ | 0.105 | 0.002 |

Only the dominant phyla with a mean relative abundance more than 1% in one group were listed.
