# Supplementary material for: Microbiome–host co-oscillation patterns in remodeling of colonic homeostasis during adaptation to a high-grain diet in a sheep model
Source: Anim Microbiome. 2020 Jul 9;2:22. doi: 10.1186/s42523-020-00041-9 (PMC7807687; doi:10.1186/s42523-020-00041-9)
Supplement: Supplementary file 6 — Additional file 6 Table S5. Serial changes in the abundance of predominant genera (% of total sequences) in colonic digesta. (Mean values with their standard errors; n = 5). [file 42523_2020_41_MOESM6_ESM.docx]

**Table S5. Serial changes in the abundance of predominant genera (% of total sequences) in colonic digesta. (Mean values with their standard errors; n = 5)**

| Phylum | Genus | CON | HG7 | HG14 | HG28 | SEM | *P* |
| --- | --- | --- | --- | --- | --- | --- | --- |
| Firmicutes | Unclassified Ruminococcaceae | 45.33^a^ | 17.72^b^ | 26.23^ab^ | 20.81^b^ | 2.873 | 0.010 |
|  | Unclassified Lachnospiraceae | 3.88 | 8.56 | 6.36 | 8.84 | 0.772 | 0.056 |
|  | Unclassified Christensenellaceae | 6.68^a^ | 1.72^b^ | 3.43^ab^ | 5.48^ab^ | 0.943 | 0.027 |
|  | *Roseburia* | 1.31 | 3.29 | 1.78 | 5.64 | 0.745 | 0.058 |
|  | *Blautia* | 1.20^b^ | 5.46^a^ | 4.45^a^ | 4.91^a^ | 0.617 | 0.013 |
|  | Unclassified vadinBB60 | 4.16^a^ | 1.00^b^ | 2.28^ab^ | 2.03^b^ | 0.380 | 0.032 |
|  | *Coprococcus* | 0.38^b^ | 1.00^ab^ | 2.72^a^ | 3.99^a^ | 0.617 | 0.005 |
|  | *Ruminococcus* | 0.50^b^ | 3.87^a^ | 1.53^ab^ | 2.19^a^ | 0.491 | 0.038 |
|  | *Oscillibacter* | 0.41^b^ | 1.46^ab^ | 3.55^a^ | 2.20^a^ | 0.357 | 0.006 |
|  | *Clostridium_sensu_stricto_1* | <0.01^b^ | 1.35^a^ | 1.03^a^ | 2.98^a^ | 0.397 | 0.007 |
|  | *Faecalibacterium* | 0.02^b^ | 0.04^b^ | 0.05^b^ | 1.89^a^ | 0.345 | 0.009 |
|  | Unclassified Erysipelotrichaceae | 0.20^b^ | 0.92^ab^ | 1.40^a^ | 1.68^a^ | 0.171 | 0.005 |
|  | *Anaerostipes* | <0.01^b^ | 1.10^a^ | 1.04^a^ | 0.84^a^ | 0.196 | 0.012 |
| Bacteroidetes | *Bacteroides* | 3.09 | 16.48 | 8.62 | 7.47 | 2.032 | 0.212 |
|  | Unclassified Prevotellaceae | 2.62 | 12.92 | 5.87 | 12.35 | 2.259 | 0.251 |
|  | *Prevotella* | 0.11^b^ | 5.72^a^ | 8.35^a^ | 2.50^ab^ | 1.656 | 0.048 |
|  | *RC9_gut_group* | 6.17^a^ | 2.54^ab^ | 3.66^ab^ | 0.56^b^ | 0.668 | 0.030 |
|  | *Alistipes* | 3.45 | 4.53 | 3.47 | 2.46 | 0.764 | 0.896 |
|  | Unclassified RF16 | 1.29 | <0.01 | 2.26 | 0.55 | 0.509 | 0.080 |
|  | *Phocaeicola* | 2.17^a^ | 0.15^bc^ | 1.34^ab^ | <0.01^c^ | 0.281 | 0.005 |
|  | Unclassified BS11_gut_group | 1.17^a^ | 0.00^b^ | 0.20^b^ | 0.10^b^ | 0.135 | 0.006 |
| Proteobacteria | *Halomonas* | 0.56^ab^ | 1.79^a^ | 0.90^ab^ | 0.18^b^ | 0.185 | 0.031 |
|  | *Campylobacter* | 1.39 | 0.75 | 1.05 | 0.47 | 0.276 | 0.484 |
| Verrucomicrobia | *Akkermansia* | 1.75^a^ | 0.18^b^ | 0.32^b^ | 0.21^b^ | 0.167 | 0.014 |
| Cyanobacteria | Unclassified Gastranaerophilales | 1.08^a^ | <0.01^b^ | 0.00^b^ | 0.20^ab^ | 0.105 | 0.001 |
| Actinobacteria | *Bifidobacterium* | <0.01^b^ | 0.61^a^ | 0.30^ab^ | 1.51^a^ | 0.234 | 0.009 |

Only the dominant genera with a mean relative abundance more than 1% in one group were listed.
