# Supplementary material for: Microbiome–host co-oscillation patterns in remodeling of colonic homeostasis during adaptation to a high-grain diet in a sheep model
Source: Anim Microbiome. 2020 Jul 9;2:22. doi: 10.1186/s42523-020-00041-9 (PMC7807687; doi:10.1186/s42523-020-00041-9)
Supplement: Supplementary file 7 — Additional file 7 Table S6. GO enrichment analysis of the M10 genes in biological processes. Only terms with p < 0.05 were listed. [file 42523_2020_41_MOESM7_ESM.docx]

**Table S6. GO enrichment analysis of the M10 genes in biological processes. Only terms with *p* < 0.05 were listed.**

| Terms | | Count | Percentage (%) | *P* | |
| --- | --- | --- | --- | --- | --- |
| GO:0009101~glycoprotein biosynthetic process | 13 | | 4.33 | <0.001 |  |
| GO:0043413~macromolecule glycosylation | | 11 | 3.67 | <0.001 | |
| GO:0006486~protein glycosylation | | 11 | 3.67 | <0.001 | |
| GO:0070085~glycosylation | | 11 | 3.67 | <0.001 | |
| GO:1901137~carbohydrate derivative biosynthetic process | | 18 | 6.00 | <0.001 | |
| GO:0009100~glycoprotein metabolic process | | 13 | 4.33 | 0.001 | |
| GO:0044723~single-organism carbohydrate metabolic process | | 17 | 5.67 | 0.001 | |
| GO:0005975~carbohydrate metabolic process | | 19 | 6.33 | 0.002 | |
| GO:0050680~negative regulation of epithelial cell proliferation | | 7 | 2.33 | 0.004 | |
| GO:1901135~carbohydrate derivative metabolic process | | 22 | 7.33 | 0.004 | |
| GO:0030968~endoplasmic reticulum unfolded protein response | | 5 | 1.67 | 0.007 | |
| GO:0050678~regulation of epithelial cell proliferation | | 10 | 3.33 | 0.008 | |
| GO:0034620~cellular response to unfolded protein | | 5 | 1.67 | 0.008 | |
| GO:0006986~response to unfolded protein | | 5 | 1.67 | 0.011 | |
| GO:0051093~negative regulation of developmental process | | 17 | 5.67 | 0.011 | |
| GO:0044712~single-organism catabolic process | | 15 | 5.00 | 0.012 | |
| GO:0035967~cellular response to topologically incorrect protein | | 5 | 1.67 | 0.013 | |
| GO:1902532~negative regulation of intracellular signal transduction | | 12 | 4.00 | 0.016 | |
| GO:0035966~response to topologically incorrect protein | | 5 | 1.67 | 0.017 | |
| GO:0006915~apoptotic process | | 27 | 9.00 | 0.020 | |
| GO:0050873~brown fat cell differentiation | | 4 | 1.33 | 0.020 | |
| GO:0007626~locomotory behavior | | 8 | 2.67 | 0.022 | |
| GO:0045815~positive regulation of gene expression, epigenetic | | 3 | 1.00 | 0.022 | |
| GO:0019752~carboxylic acid metabolic process | | 15 | 5.00 | 0.023 | |
| GO:0043436~oxoacid metabolic process | | 15 | 5.00 | 0.024 | |
| GO:0043114~regulation of vascular permeability | | 3 | 1.00 | 0.026 | |
| GO:0006631~fatty acid metabolic process | | 8 | 2.67 | 0.027 | |
| GO:0008219~cell death | | 29 | 9.67 | 0.028 | |
| GO:0010771~negative regulation of cell morphogenesis involved in differentiation | | 5 | 1.67 | 0.035 | |
| GO:1901215~negative regulation of neuron death | | 6 | 2.00 | 0.035 | |
| GO:0006629~lipid metabolic process | | 20 | 6.67 | 0.035 | |
| GO:0070127~tRNA aminoacylation for mitochondrial protein translation | | 2 | 0.67 | 0.036 | |
| GO:2000169~regulation of peptidyl-cysteine S-nitrosylation | | 2 | 0.67 | 0.036 | |
| GO:0034976~response to endoplasmic reticulum stress | | 7 | 2.33 | 0.037 | |
| GO:0010719~negative regulation of epithelial to mesenchymal transition | | 3 | 1.00 | 0.037 | |
| GO:0012501~programmed cell death | | 27 | 9.00 | 0.039 | |
| GO:0051241~negative regulation of multicellular organismal process | | 19 | 6.33 | 0.039 | |
| GO:0006464~cellular protein modification process | | 44 | 14.67 | 0.039 | |
| GO:0036211~protein modification process | | 44 | 14.67 | 0.039 | |
| GO:0008285~negative regulation of cell proliferation | | 13 | 4.33 | 0.039 | |
| GO:0030336~negative regulation of cell migration | | 7 | 2.33 | 0.039 | |
| GO:0097006~regulation of plasma lipoprotein particle levels | | 4 | 1.33 | 0.040 | |
| GO:0032787~monocarboxylic acid metabolic process | | 10 | 3.33 | 0.044 | |
| GO:0008283~cell proliferation | | 28 | 9.33 | 0.046 | |
| GO:0060997~dendritic spine morphogenesis | | 3 | 1.00 | 0.046 | |
| GO:0018198~peptidyl-cysteine modification | | 3 | 1.00 | 0.046 | |
| GO:0048193~Golgi vesicle transport | | 6 | 2.00 | 0.046 | |
| GO:0060284~regulation of cell development | | 16 | 5.33 | 0.046 | |
| GO:0060688~regulation of morphogenesis of a branching structure | | 4 | 1.33 | 0.048 | |
| GO:2000146~negative regulation of cell motility | | 7 | 2.33 | 0.048 | |
