# Supplementary material for: Microbiome–host co-oscillation patterns in remodeling of colonic homeostasis during adaptation to a high-grain diet in a sheep model
Source: Anim Microbiome. 2020 Jul 9;2:22. doi: 10.1186/s42523-020-00041-9 (PMC7807687; doi:10.1186/s42523-020-00041-9)
Supplement: Supplementary file 8 — Additional file 8 Table S7. The expression of hub genes was enriched in the M10 module. [file 42523_2020_41_MOESM8_ESM.docx]

**Table S7. The expression of hub genes was enriched in the M10 module.**

| Genus | CON | HG7 | HG14 | HG28 | SEM |
| --- | --- | --- | --- | --- | --- |
| RRBP1 | 162.56 | 183.36 | 155.63 | 160.92 | 5.630 |
| FAM217B | 1.55 | 1.22 | 1.40 | 1.61 | 0.100 |
| LOC101123184 | 2.85 | 2.48 | 2.64 | 2.80 | 0.100 |
| FGFRL1 | 1.73 | 2.79 | 2.22 | 2.02 | 0.180 |
| NCK2 | 29.35 | 37.05 | 28.28 | 31.76 | 1.364 |
| CD68 | 26.75 | 17.53 | 22.29 | 23.19 | 1.208 |
| MTERF2 | 5.60 | 4.83 | 5.49 | 5.61 | 0.123 |
| DFNB31 | 1.63 | 2.87 | 2.21 | 1.84 | 0.228 |
| PPIC | 25.31 | 40.32 | 33.93 | 32.65 | 2.557 |
| RMND5B | 11.55 | 13.39 | 11.74 | 12.01 | 0.399 |
| MTCH1 | 92.24 | 113.16 | 100.28 | 94.07 | 2.515 |
| RGS12 | 0.90 | 1.33 | 1.08 | 1.09 | 0.067 |
| KIAA1161 | 7.71 | 12.70 | 10.01 | 10.71 | 0.604 |
| DAP | 39.46 | 42.01 | 38.65 | 39.28 | 0.951 |
| ANGPT4 | 2.39 | 1.93 | 2.20 | 2.31 | 0.119 |
| CTSA | 82.48 | 68.53 | 78.12 | 81.26 | 2.361 |
| RAP1GAP | 20.87 | 30.90 | 30.97 | 33.88 | 2.069 |
| SRM | 10.39 | 12.79 | 12.90 | 10.03 | 0.681 |
| RAD51C | 4.49 | 3.51 | 4.34 | 3.40 | 0.178 |
| SET | 54.17 | 45.11 | 50.80 | 52.95 | 2.239 |
| PDK1 | 2.38 | 2.51 | 2.04 | 2.09 | 0.112 |
| TNFRSF21 | 36.14 | 55.15 | 34.69 | 53.42 | 3.502 |
| QPCTL | 6.07 | 7.36 | 6.94 | 7.18 | 0.203 |
| C12H1orf27 | 4.61 | 4.35 | 4.87 | 4.90 | 0.147 |
| BTBD9 | 9.91 | 11.69 | 11.08 | 11.47 | 0.272 |
| LOC101102627 | 3.19 | 4.40 | 4.12 | 3.80 | 0.360 |
| P2RY14 | 1.31 | 0.91 | 1.55 | 1.34 | 0.088 |
| CMAS | 58.14 | 70.87 | 55.31 | 58.77 | 2.932 |
| BCAR1 | 43.38 | 61.47 | 54.77 | 49.98 | 2.841 |
| LOC106991690 | 4.12 | 5.73 | 6.68 | 5.94 | 0.741 |
| LOC106991303 | 12.77 | 16.72 | 13.47 | 12.81 | 0.444 |
| B3GNT6 | 22.58 | 30.39 | 19.53 | 19.61 | 1.904 |
| PTGER4 | 4.74 | 12.19 | 14.48 | 5.28 | 1.431 |
| ZDHHC5 | 18.65 | 26.46 | 20.25 | 20.01 | 1.098 |
| CIDEC | 0.65 | 2.23 | 0.70 | 2.00 | 0.394 |
| LGMN | 57.58 | 38.48 | 46.70 | 44.64 | 2.657 |
