# Supplementary material for: Microbiome–host co-oscillation patterns in remodeling of colonic homeostasis during adaptation to a high-grain diet in a sheep model
Source: Anim Microbiome. 2020 Jul 9;2:22. doi: 10.1186/s42523-020-00041-9 (PMC7807687; doi:10.1186/s42523-020-00041-9)
Supplement: Supplementary file 9 — Additional file 9 Table S8. Ingredient and chemical composition of the diet (dry matter basis). [file 42523_2020_41_MOESM9_ESM.docx]

**Table S8. Ingredient and chemical composition of the diet (dry matter basis).**

| Items | Diet | |
| --- | --- | --- |
|  | CON | HG |
| Ingredient composition, % DM |  |  |
| Oat hay | 63.40 | 26.00 |
| Alfalfa hay | 33.00 | 14.00 |
| Corn meal | 0.00 | 34.20 |
| Wheat meal | 0.00 | 18.00 |
| Soybean meal | 0.00 | 4.20 |
| CaCO3 | 1.00 | 1.00 |
| NaCl, salt | 0.40 | 0.40 |
| Calcium phosphate dibasic | 1.20 | 1.20 |
| Mineral and vitamin supplement^1^ | 1.00 | 1.00 |
| Nutrient composition^2^ |  |  |
| DE, MJ/kg DM | 8.88 | 11.73 |
| Crude protein, % DM | 11.18 | 11.92 |
| Crude fat, % DM | 2.09 | 2.49 |
| Crude fiber, % DM | 28.41 | 12.90 |
| Neutral detergent fiber, %DM | 44.45 | 24.54 |
| Acid detergent fiber, % DM | 19.52 | 10.15 |
| Crude ash, % DM | 8.34 | 4.53 |
| Starch, % DM | 3.25 | 32.34 |

^1^ Contained 16% calcium carbonate, 102 g/kg of Zn, 47 g/kg of Mn, 26 g/kg of Cu, 1,140 mg/kg of I, 500 mg/ke of Se, 340 mg/kg of Co, 17,167,380 IU/kg of Vitamin A, 858,370 IU/kg of vitamin D, and 23,605 IU/kg of vitamin E.

^2^ Values were analyzed based on the Chinese Feed Database (2015).
