# Supplementary material for: Microbiome–host co-oscillation patterns in remodeling of colonic homeostasis during adaptation to a high-grain diet in a sheep model
Source: Anim Microbiome. 2020 Jul 9;2:22. doi: 10.1186/s42523-020-00041-9 (PMC7807687; doi:10.1186/s42523-020-00041-9)
Supplement: Supplementary file 10 — Additional file 10 Table S9. The primer sequences of genes related to cell apoptosis in the colonic epithelium for qRT-PCR. [file 42523_2020_41_MOESM10_ESM.docx]

**Table S9. The primer sequences of genes related to cell apoptosis in the colonic epithelium for qRT-PCR.**

| Gene Name | Gene ID | Primer sequence (5’→3’) | Amplicon Size (bp) |
| --- | --- | --- | --- |
| Caspase-3 | XM_015104559.1 | For: CAGCTACCTCAAACACAGTTGG | 203 |
|  |  | R: TGATACAGTGGCATACCCACAT |  |
| Caspase-8 | NC_019459.2 | For: TCCAGGATTCGCCTCTGGTA | 133 |
|  |  | R: CCGGCTTAGGAACTTGAGGG |  |
| Bcl-2 | XM_012103831.2 | For: GTGGATGACCGAGTACCTGAAC | 197 |
|  |  | R: CTTCACTTATGGCCCAGATAGG |  |
| Bax | XM_015100640.1 | For: TGTCCTCCCCCAGAGATCAG | 97 |
|  |  | R: GGGCCCTAGAGGAGAAAGGA |  |
| Bad | NXM_004019650.3 | For: TTTCGGAAGACTGAGGTCTGAT | 185 |
|  |  | R: CGGCGAAGTTAGGGTTAATCTC |  |
